# Supplementary material for: Majority sensing in synthetic microbial consortia
Source: Nat Commun. 2020 Jul 21;11:3659. doi: 10.1038/s41467-020-17475-z (PMC7374166; doi:10.1038/s41467-020-17475-z)
Supplement: Supplementary file 1 — Supplementary Information [file 41467_2020_17475_MOESM1_ESM.pdf]

# Supplementary Information

# Majority sensing in synthetic microbial consortia

Alnahhas et al.

## Supplementary Methods

These methods expand upon the mathematical model presented in the main text. The majority wins circuits are shown in the main text in Figure 1. In our mathematical model of the consortium, we describe the dynamics of fluorescence, as well as the growth of the two strains within the population. We model the size of the total population using the logistic equation,

$$\dot{N} = \lambda N\Big(1 - \frac{N}{C}\Big), \tag{1}$$

where $N$ denotes the total number of cells in the liquid culture, $\lambda$ is the cell growth rate coefficient, and $C$ denotes the carrying capacity of the container. The logistic growth model is appropriate for cells growing in a liquid culture: The population goes through a period of exponential growth, followed by a saturation phase wherein cells run out of nutrients and start dying or using secondary carbon sources. In this second phase, cell growth rate slows and tends to zero, while the cell count approaches the carrying capacity $C$. (See experimental data showing the optical density (OD) of the cells in the container in Supplementary Figure 3). Cell growth dynamics in the saturation phase is complicated. We did not model saturation phase growth dynamics in detail, as we took our experimental measurements during the exponential phase.

The consortium is composed of two strains, and therefore $N = N_1 + N_2$, where $N_1$ and $N_2$ are the number of yellow and blue cells, respectively. By setting $r = N_1/N$ (the ratio of yellow strain cell count to total cell count), we have $N_1 = rN$ and $N_2 = (1-r)N$. Here we assume that the ratio $r$ remains constant throughout the experiment at the value we set when we mix the strains in the beginning of the experiment. We verified this assumption by counting the number of cells in the yellow and blue colonies that were taken from the liquid culture and plated on agar plates (See Supplementary Fig. 5).

We model the dynamics of the protein synthesis and degradation in cells with the following ODEs,

$$\dot{x}_1 = \alpha_0 \frac{1}{1 + \left(\frac{(1-r)Nx_2}{\theta_1} h(I)\right)^{n_1} + L_1 h(I)} - \beta x_1 - \frac{D_1 x_1}{Q + x_1} \tag{2}$$

$$\dot{x}_2 = \alpha_0 \frac{1}{1 + \left(\frac{rNx_1}{\theta_2}\right)^{n_2} + L_2} - \beta x_2 - \frac{D_2 x_2}{Q + x_2} \tag{3}$$

where

$$h(I) = \frac{1}{1 + (\frac{I}{K})^{\ell}} \tag{4}$$

$$\beta = \lambda\Big(1 - \frac{N}{C}\Big) \tag{5}$$

Supplementary Equations (2) and (3) describe the dynamics of the protein synthesis and degradation in the cells. In particular, $x_i$ denotes the concentration per cell of both synthase and fluorescent protein in strain $i$ ($i = 1, 2$). We assume that synthase concentration per cell equals fluorescent protein concentration per cell within each strain because the same promoters and quorum sensing molecules regulate the production of both synthase and fluorescent protein. While this assumption is wrong within individual cells due to random fluctuations, it is approximately correct when averaging over the population.

The model includes protein loss due to both dilution and enzymatic degradation. The terms $-\beta x_i$ ($i = 1, 2$) model protein dilution due to cell growth. Here $\beta$ is the instantaneous cell growth rate that satisfies $\dot{N} = \beta N$ and is given explicitly by Supplementary Equation (5). The term $\frac{D_i x_i}{Q + x_i}$ represents enzymatic degradation of protein $x_i$ ($i = 1, 2$). We obtain this term by using Michaelis-Menten dynamics for the enzymatic degradation mechanism. The parameter $D_i$ is the degradation rate coefficient for protein $x_i$, and $Q$ is the Michaelis-Menten constant.

The protein synthesis rate for strain 1 (the yellow strain) is given as a product of the maximal production rate, $\alpha_0$, and a regulatory function that takes into account the effects of the inhibition by the quorum sensing molecule, the inducer, and the leakiness. In particular, the effect of the inducer is modeled by a Hill function given in Supplementary Equation (4), with parameters $\ell$ and $K$. In the absence of inducer, *i.e.* when $I = 0$, we have $h(I) = 1$. Hence in the absence of inducer, the synthesis rate of protein $x_1$ is $(1 + L_1)^{-1}$ in the absence of the quorum sensing signal, and $\Big(1 + \Big(\frac{(1-r)Nx_2}{\theta_1}\Big)^{n_1} + L_1\Big)^{-1}$ when the quorum sensing signal is present.

We assume that the total quorum sensing signal produced by strain 2 is proportional to the overall protein concentration in strain 2, *i.e.* $(1 - r)Nx_2$, and the proportionality constant is absorbed in the parameter $\theta_1$. The parameters $n_i$ and $\theta_i$ parametrize the Hill function that represents the effect of the quorum sensing signal on the protein synthesis rate in strain $i$ ($i = 1, 2$). We assume that the inducer can overwhelm the effects of leakiness and the quorum sensing signal. Precisely, as $I$ gets large, $h(I)$ tends to zero, and the protein synthesis rate in strain 1 tends to its maximum, $\alpha_0$.

We model protein synthesis rate in strain 2 in an equivalent way. Since the two strains use similar promoters, we assume that the maximal production rate is the same in both strains. We opted not to model

the effect of inducer on strain 2 because doing so would add no new information due to the symmetry of the system.

The parameter values we used in Supplementary Equations (1)-(5) are given in Table 1. As we were only interested in getting insight about the dynamical behavior of the consortium, we did not use a systematic fitting technique to infer parameter values from experimental data. Rather, we chose biologically feasible values that lead to dynamics that closely followed those we observed experimentally. In particular, we chose the values of $\lambda$ and $C$ in Supplementary Equation (1) in a way that the cell growth dynamics closely mimics the OD data given in Supplementary Figure 3 (until around 5 hours). We chose the remaining parameters so that our experimental observations in Supplementary Figure 1 and Supplementary Figure 2 were captured. For instance, in Supplementary Figure 2, we have the ratio of the total fluorescence in induced media to that in uninduced (blank) media. Based on this information, we can find the values of the leakiness constants $L_1$ and $L_2$ (given that the rest of the parameters are fixed).

Supplementary Table 1: Parameters in the model described by Supplementary Equations (1)-(5)

| **Parameter** | **Value** |
|---:|---|
| $\lambda$ | 0.022 $\text{min}^{-1}$ |
| $C$ | 1.2 a.u. |
| $\alpha_0$ | 500 $\text{min}^{-1}$ |
| $D_i$ | 100 $\text{min}^{-1}$, $i = 1, 2$ |
| $Q$ | $3 \times 10^4$ proteins $\times$ (unit cell volume)$^{-1}$ |
| $n_i$ | 2, $i = 1, 2$ |
| $\ell$ | 2 |
| $K$ | $10^{-1}$ nM |
| $\theta_1$ | $0.46 \times 10^3$ units of protein count |
| $\theta_2$ | $1.0 \times 10^3$ units of protein count |
| $L_1$ | 0.89 |
| $L_2$ | 1.23 |

Supplementary Figure 1 shows simulation results of the model given in Supplementary Equation (1–5) corresponding to the experimental data shown in Figure 3 of the main text. In the five panels of Supplementary Figure 1, the relative fluorescence values of the yellow and blue strains are shown as a function of the yellow strain fraction, $r_\text{Y}$, at 5 different IPTG concentrations. To mimic the experimental data, the relative fluorescence values obtained by the mathematical model are calculated at a time when $N(t)$ in Supplementary Equation (1) reaches the proximity of $C$. In particular, the fluorescence values are computed for the time $t_\text{s}$ where $N(t_\text{s}) = 0.85C = (0.85)(1.2) = 1.02$, which yields $t_\text{s} = 296$ min $\approx 5$ hours. In the experiments, the fluorescence measurements were obtained approximately at the start of the cell saturation phase, that is

around 5 hours after the start of the experiment, when OD becomes almost equal to 1.

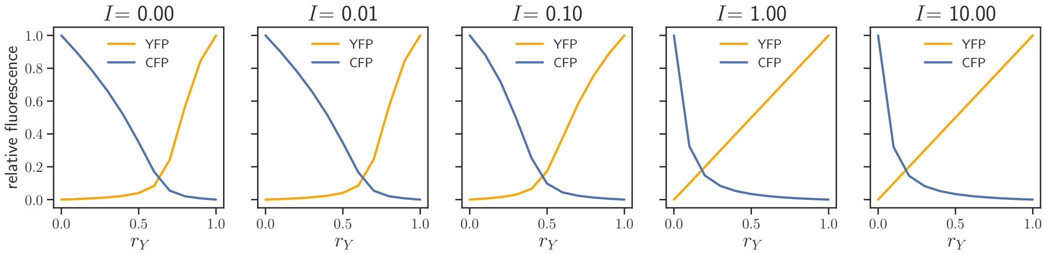


Supplementary Figure 1: **The effect of adding inducer on the majority wins pattern.** In the model, as inducer concentration increases, the amount of yellow strain needed to 'win' decreases.

We developed a similar mathematical model for the minority wins pattern that is shown in Figure 5a in the main text. As in the model for the majority wins consortium, we have one equation for the cell population dynamics while other equations model protein dynamics. However, note that in the minority wins circuit the synthase and the fluorescent protein dynamics are not directly linked. Unlike the majority wins circuit, the corresponding promoters are different, and are regulated differently by the quorum sensing signal. To capture this in our model, we use separate equations for the synthase and the fluorescent proteins in each strain. Therefore, the mathematical model for the minority wins pattern has the form

$$\dot{N} = \lambda N(1 - \frac{N}{C}) \tag{6}$$

$$\dot{x}_1 = \alpha_1 \frac{1}{1 + \left(\frac{(1-r)Nx_2}{\theta_1}\right)^{n_1}} - \beta x_1 - \frac{D_1 x_1}{Q + x_1} \tag{7}$$

$$\dot{p}_1 = \alpha_{\mathrm{p}1} \frac{\left(\frac{(1-r)Nx_2}{Z_1}\right)^{m_1}}{1 + \left(\frac{(1-r)Nx_2}{Z_1}\right)^{m_1}} - \beta p_1 - \frac{D_{\mathrm{p}1} p_1}{Q + p_1} \tag{8}$$

$$\dot{x}_2 = \alpha_2 \frac{1}{1 + \left(\frac{rNx_1}{\theta_2}\right)^{n_2}} - \beta x_2 - \frac{D_2 x_2}{Q + x_2} \tag{9}$$

$$\dot{p}_2 = \alpha_{\mathrm{p}2} \frac{\left(\frac{rNx_1}{Z_2}\right)^{m_2}}{1 + \left(\frac{rNx_1}{Z_2}\right)^{m_2}} - \beta p_2 - \frac{D_{\mathrm{p}2} p_2}{Q + p_2} \tag{10}$$

Supplementary Equation (6) describes the cell population dynamics, as in the earlier model. Supplementary Equations (7) and (9) describe the synthase dynamics, while Supplementary Equations (8) and (10) describe the dynamics of the fluorescent protein. In particular, $x_1$, $x_2$, $p_1$, and $p_2$ are cellular concentrations of rhlI, cinI, sfYFP, and sfCFP, respectively. The parameters $\alpha_1$ and $\alpha_2$ are the synthesis rates of the corresponding synthases and $\alpha_{\mathrm{p}1}$ and $\alpha_{\mathrm{p}2}$ are the synthesis rates of the fluorescent proteins. The parameters

$n_i$, $\theta_i$, $i = 1, 2$, are the Hill function parameters that represent the inhibition of the synthase production by quorum sensing signal and $m_i$, $Z_i$, $i = 1, 2$, are the Hill function parameters that represent the activation of the fluorescent protein production by quorum sensing signal. Note that the quorum sensing signal is proportional to the total amount of the synthase proteins in each strain. For example, the quorum sensing signal from strain 2 to strain 1 is proportional to $(1 - r)Nx_2$. As in the case of the majority wins circuit, we included dilution terms and enzymatic degradation terms. However, in the case of the minority wins circuit we do not model the effect of leakiness, as it had only a minor effect on the behavior of the model. In addition, we did not incorporate the effect of inducer in the model. Such effects could be easily included.

We used the parameter values given in Table 2 for the minority wins model.

Supplementary Table 2: Parameters in the model described by Supplementary Equations (6)-(10).

| **Parameter** | **Value** |
|---:|---|
| $\lambda$ | 0.022 $\text{min}^{-1}$ |
| $C$ | 1.2 a.u. |
| $\alpha_1$, $\alpha_2$ | 28 $\text{min}^{-1}$ |
| $\alpha_{\text{p1}}$ | 72 $\text{min}^{-1}$ |
| $\alpha_{\text{p2}}$ | 28 $\text{min}^{-1}$ |
| $D_1, D_2, D_{\text{p1}}, D_{\text{p2}}$ | 100 $\text{min}^{-1}$, $i = 1, 2$ |
| $Q$ | $3 \times 10^4$ proteins $\times$ (unit cell volume)$^{-1}$ |
| $n_1, n_2, m_1, m_2$ | 2 |
| $\theta_1, \theta_2, Z_1, Z_2$ | $10^3$ units of protein count |

# Supplementary Figures

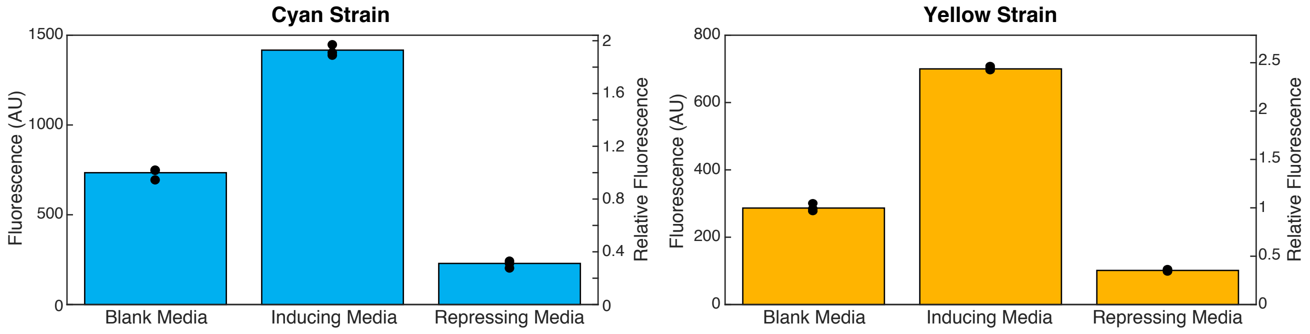


Supplementary Figure 2: **Fluorescence state of majority wins strains in monoculture.** Each graph shows the (background subtracted) fluorescence level of each strain in monoculture with no inducer or repressor ('Blank Media'), with 10mM of that strain's inducer (ribose for cyan strain and IPTG for yellow strain, 'Inducing Media'), or with conditioned media with the opposite strain's QS molecule ('Repressing Media'). In both graphs, the fluorescence was ON in the blank media, but to a lesser extent than in the inducing media. This is due to slight leaky expression of the repressors in both strains. The presence of the opposite strain's QS molecule in the repressing media reduced fluorescence significantly. This is as expected for the majority wins strains The repressing media was media in which the opposite strain was previously grown in then we spun down the cells to only carry over the media and QS molecule. n=3 biological replicates shown as black dots.

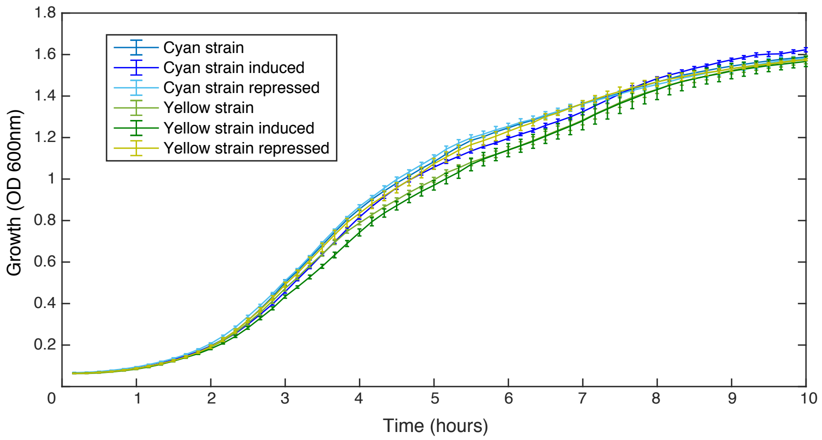


Supplementary Figure 3: **Growth curves of strains in monoculture.** In shades of blue are the growth of the cyan strain grown in monoculture over time in blank media, inducing media (10mM ribose), and repressing media (conditioned media with the yellow strain's QS molecule). In shades of green are the growth of the yellow strain grown in monoculture over time in blank media, inducing media (10mM IPTG), and repressing media (conditioned media with the cyan strain's QS molecule). These are from the same experiment used for the data presented in Supplementary Fig. 2. There is no substantial difference between the growth rates. The red dashed line represents the general time point from which the data for all graphs in the main text was taken. n=3 biological replicates; error bars display standard deviation.

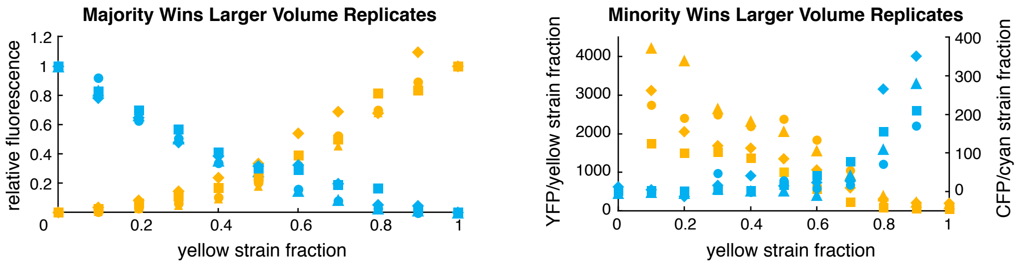


Supplementary Figure 4: **Larger population replicates.** Left graph shows fluorescence intensity of the majority wins consortium in larger, 2ml volumes grown in a 24 deep well plate (n=4). 200 microliter samples were taken from the deep well plate to measure in the plate reader at the same OD as all other experiments in the main text. Results were obtained using 10% increments starting with 100% cyan strain and ending with 100% yellow strain. To compare fluorescence intensities with a maximum, they are normalized to the values measured in wells containing a single strain of the respective color (100% wells). Fluorescence intensity still correlates with strain fraction in a majority wins pattern at this larger volume. Same symbols with different colors represents the yellow and cyan intensities from the same well of the same replicate experiment. Right graph shows fluorescence intensity of the minority wins consortium in larger, 2ml volumes grown in a 24 deep well plate (n=4). 200 microliter samples were taken from the deep well plate to measure in the plate reader at the same OD as all other experiments in the main text. Results were obtained using 10% increments starting with 100% cyan strain and ending with 100% yellow strain. Data is normalized to strain fraction to account for any change in fluorescence due to the decrease in the number of cells containing the fluorescence gene. Fluorescence intensity still correlates with strain fraction in a minority wins pattern at this larger volume. Same symbols with different colors represents the yellow and cyan intensities from the same well of the same replicate experiment.

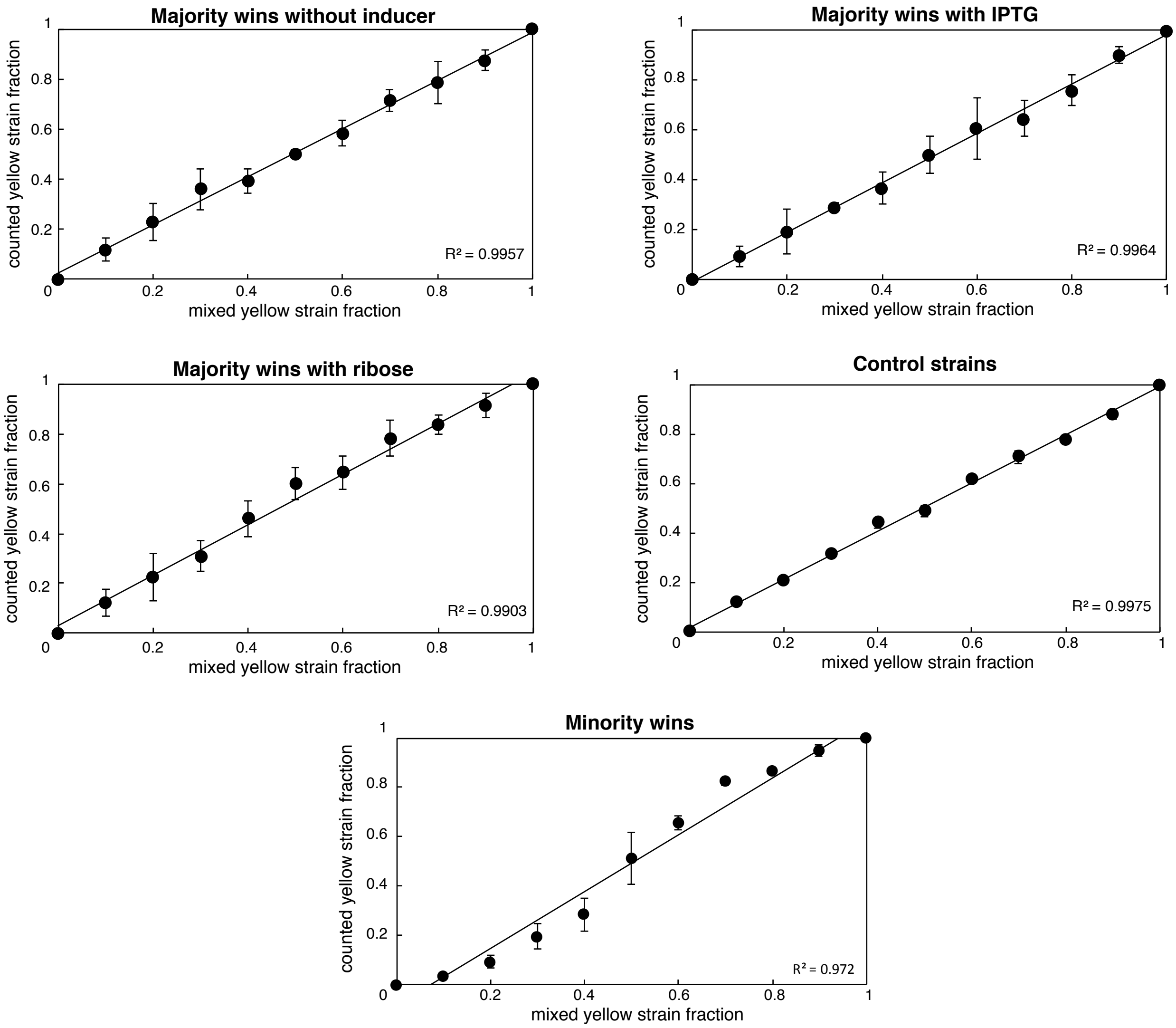


Supplementary Figure 5: **Measuring strain ratios.** To confirm that actual strain ratios were the same as the mixed strain ratios, cultures were serially diluted and plated. Cyan and yellow colonies were counted to determine strain fractions. Graphs show counted versus mixed yellow strain fractions for a subset of replicates from each of the experiments from the main text. For each graph, n=4 biological replicates with the mean shown as dots and error bars displaying standard deviation. Linear fit lines are displayed with R-squared values given. In all cases, the measured strain ratios very closely match the mixed strain ratios.

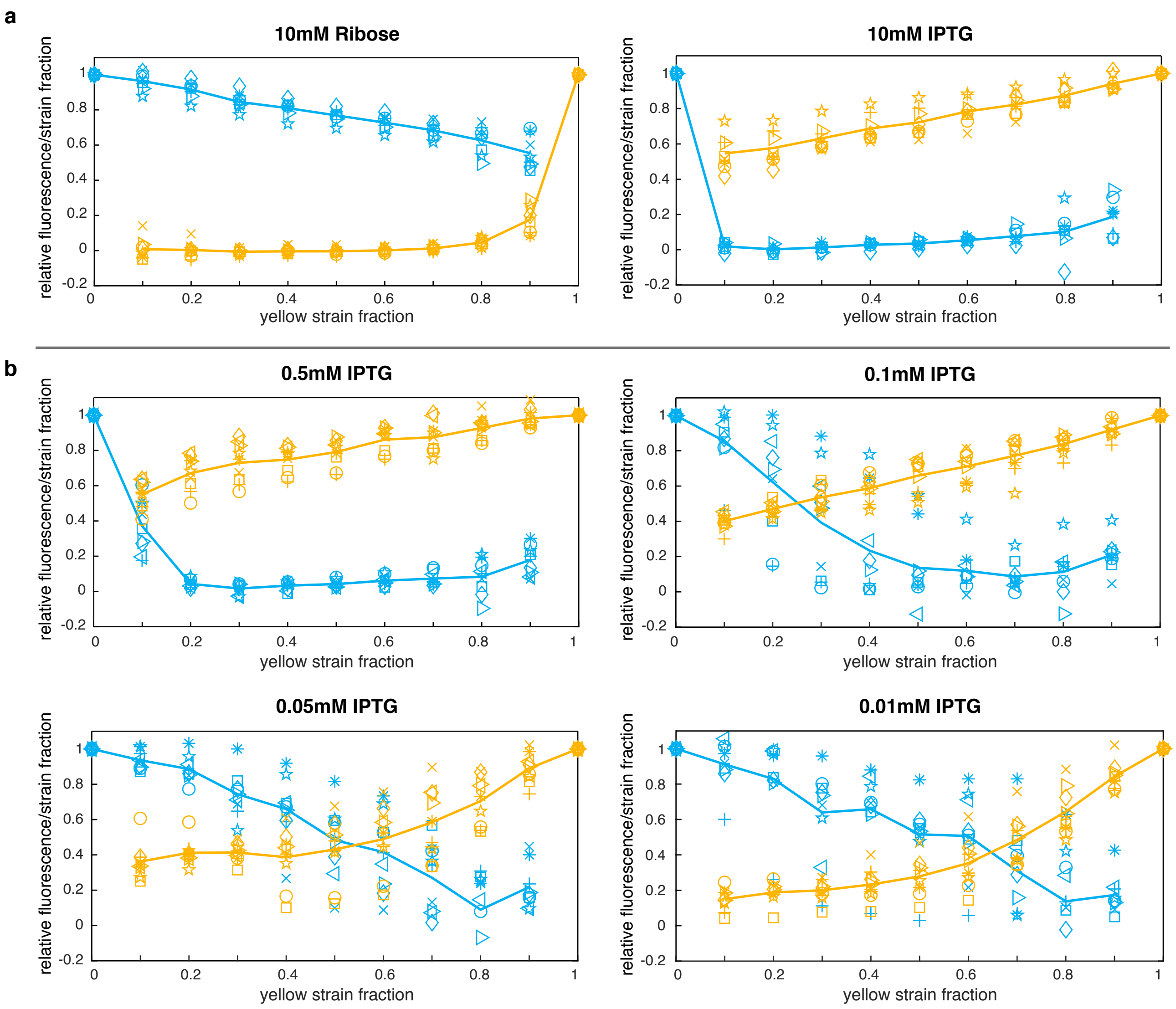


Supplementary Figure 6: **Inducer effect on the majority wins pattern - normalized to strain ratio.** **(a)** Relative cyan (blue curve) and yellow (yellow curve) fluorescence as a function of strain fraction in the presence of 10mM ribose (left) or 10mM IPTG (right) (n=9). Full induction with 10mM ribose or IPTG induces the respective strain regardless of strain fraction. Fluorescences are normalized to maximum values from wells containing a single strain of the respective color (100% wells) and normalized to strain fraction to account for any change in fluorescence due to the decrease in the number of cells containing the fluorescence gene. **(b)** Decreasing amounts of IPTG shift the fluorescence curves differently and alter the majority wins pattern less (n=9). See Supplementary Fig. 1 for corresponding modeling data showing the modulation of cross over point with inducer. For each of the above graphs, similar symbols with different colors represent the yellow and cyan intensities from the same well of the same replicate experiment and lines represent averages across all replicates. Further, fluorescences are normalized to maximum values from wells containing a single strain of the respective color (100% wells) and normalized to strain fraction to account for any change in fluorescence due to the decrease in the number of cells containing the fluorescence gene.

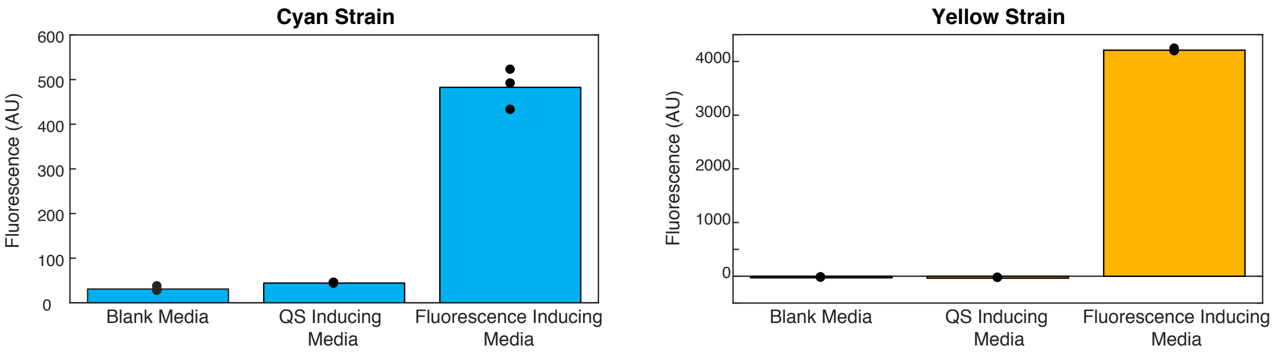


Supplementary Figure 7: **Fluorescence state of minority wins strains in monoculture.** Each graph shows the (background subtracted) fluorescence level of each strain in monoculture without external inducer or QS molecules for the 'Blank Media', with 10mM of ribose or IPTG for the 'QS Inducing Media' of the cyan and yellow strains respectively, or with conditioned media containing the opposite strain's QS molecule for the 'Fluorescence Inducing Media'. In both graphs, the fluorescence was OFF in the blank media and the QS inducing media. The presence of the opposite strain's QS molecule in the fluorescence inducing media increased fluorescence significantly. This is as expected for the minority wins strains. The fluorescence inducing media was media in which the opposite strain was previously grown in then we spun down the cells to only carry over the media and QS molecule. n=3 biological replicates shown as black dots.

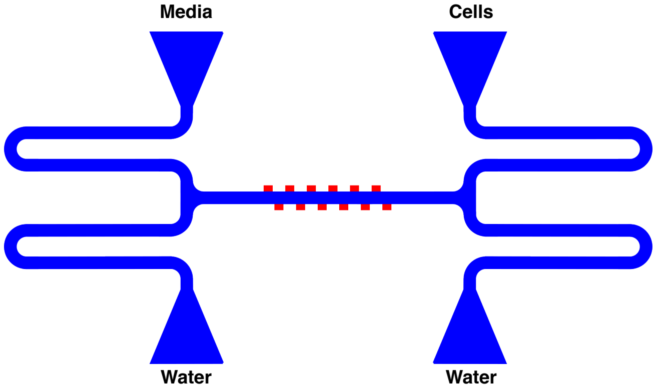


Supplementary Figure 8: **Microfluidic device.** In blue are the 10 µm tall media flow channels, and in red are the 1.5 µm tall cell trapping channels. Blue triangles are ports to which media, cells, and water reservoirs are connected. Water reservoirs serve as waste uptake.

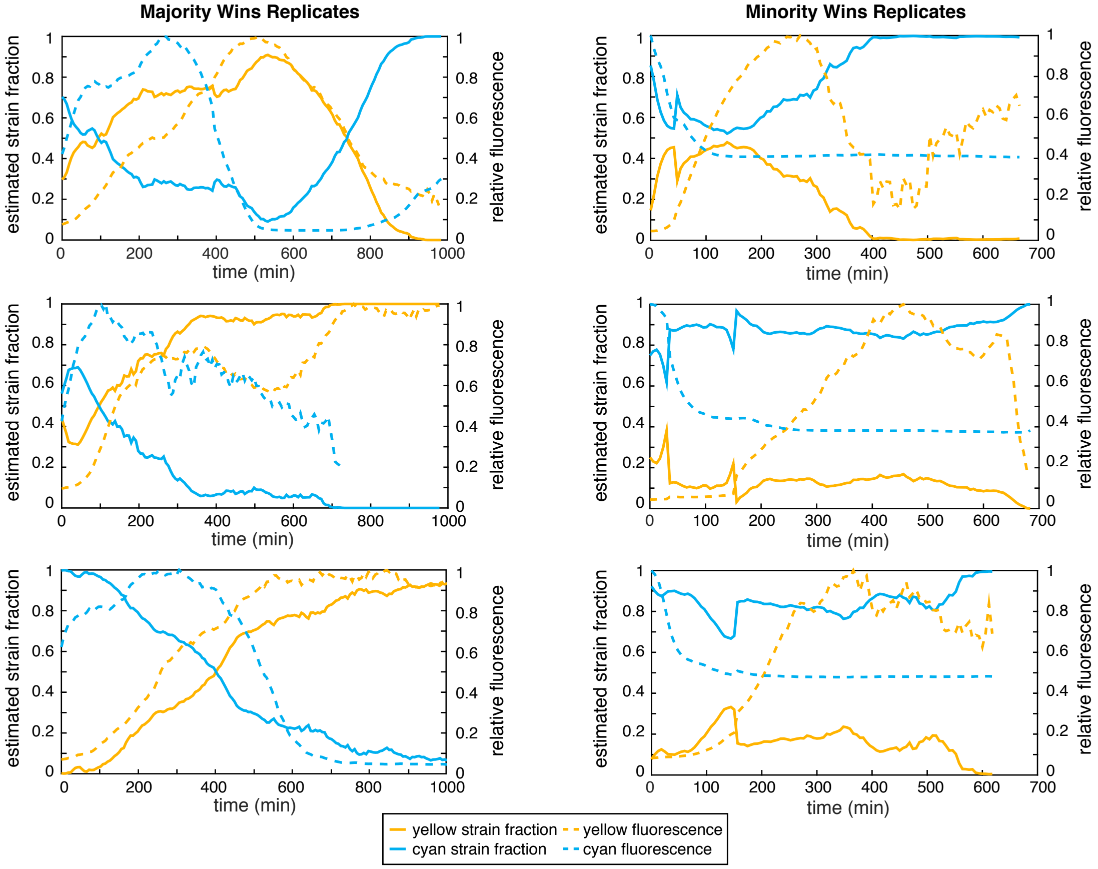


Supplementary Figure 9: **Microfluidic replicates.** More replicates of the data presented in Fig. 6. For the majority wins consortium, the fluorescence patterns follows the strain ratio patterns, and for the minority wins consortium, the fluorescence pattern are opposite of the strain ratio patterns. Top to bottom represent data from Supplemental videos 3-5 (left) and Supplemental Videos 6-8 (right).

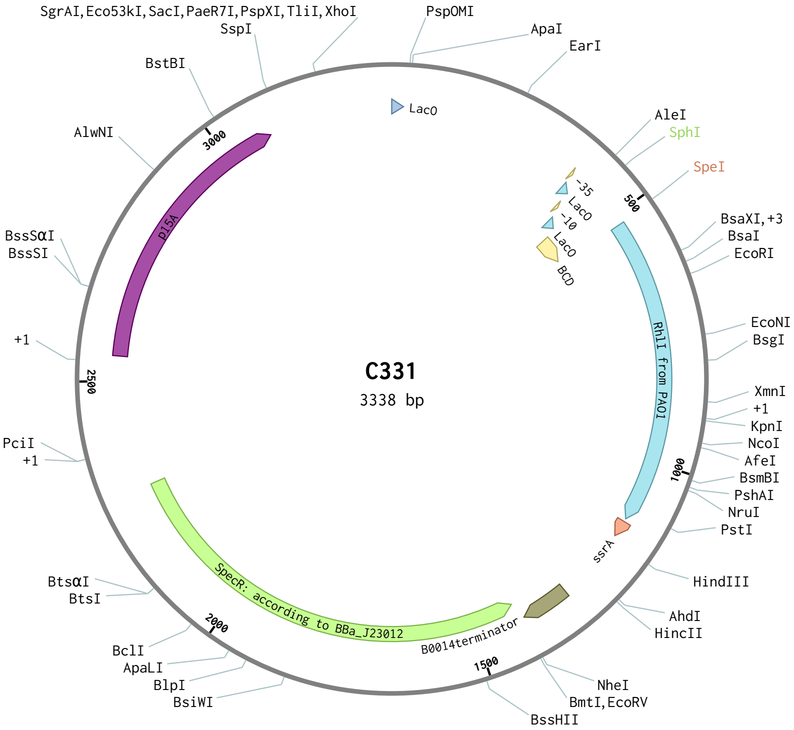


Supplementary Figure 10: **Map of plasmid C331.** QS plasmid for the majority wins cyan strain/minority wins yellow strain. See Supplementary Tables 3 and 4 below for details.

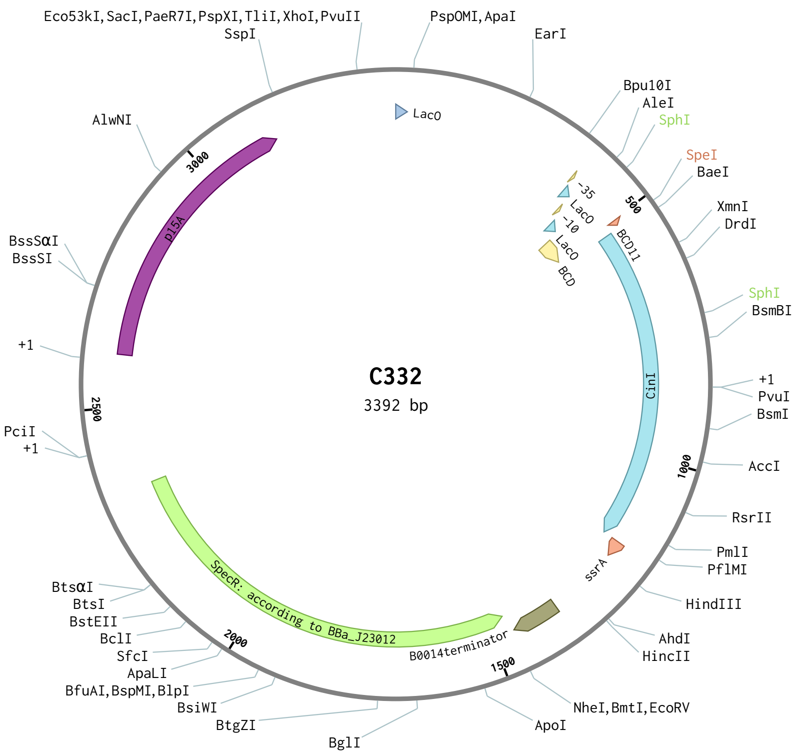


Supplementary Figure 11: **Map of plasmid C332.** QS plasmid for the majority wins yellow strain/minority wins cyan strain. See Supplementary Tables 3 and 4 below for details.

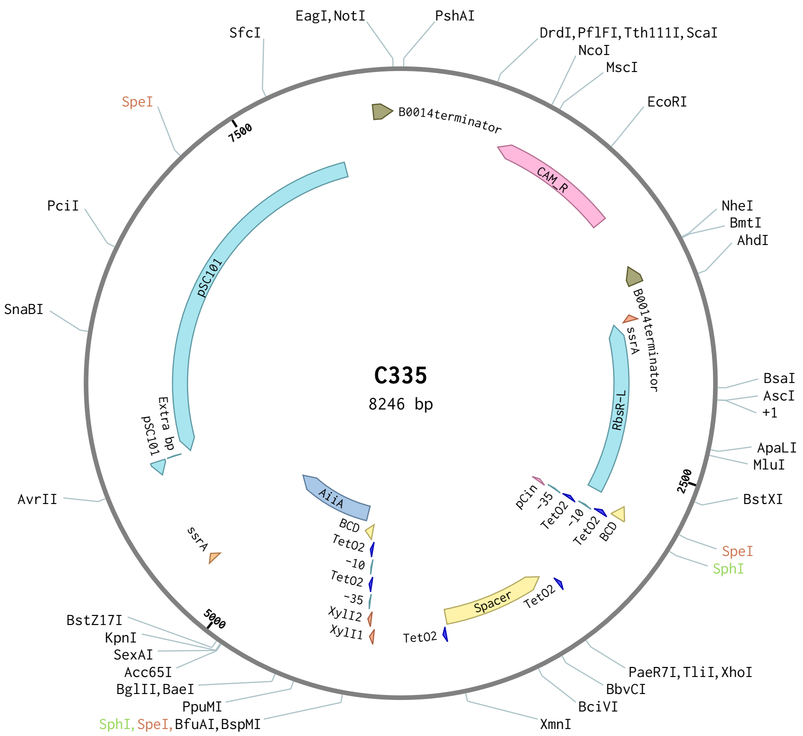


Supplementary Figure 12: **Map of plasmid C335.** Repressor plasmid for the majority wins cyan strain/minority wins yellow strain. See Supplementary Tables 3 and 4 below for details..

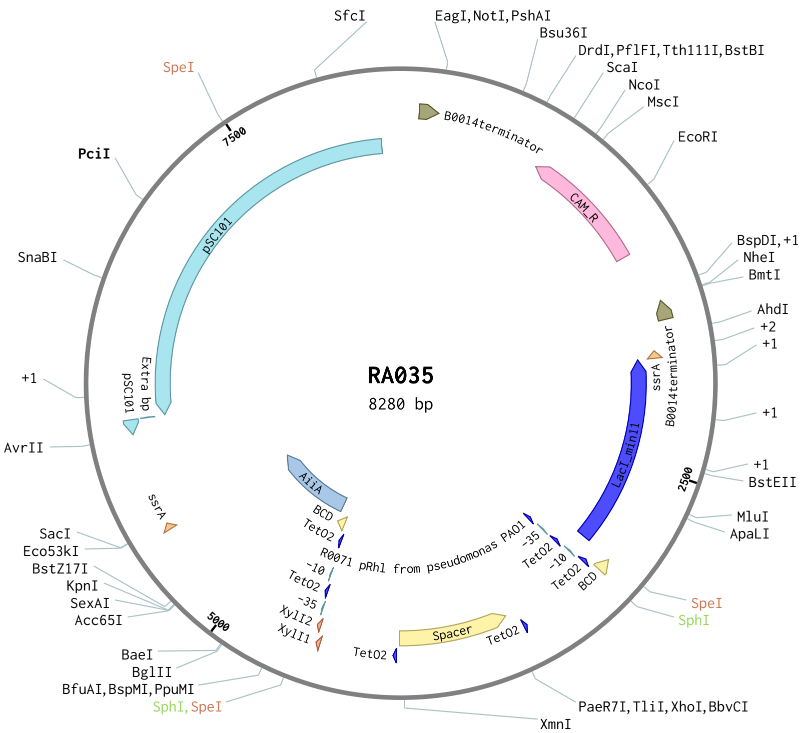


Supplementary Figure 13: **Map of plasmid RA035.** Repressor plasmid for the majority wins yellow strain/minority wins cyan strain. See Supplementary Tables 3 and 4 below for details.

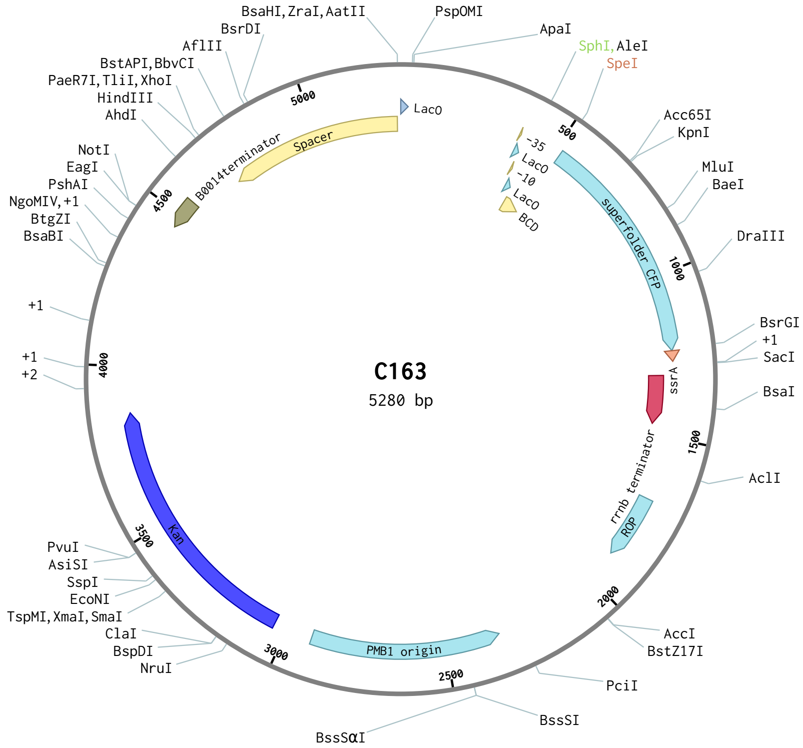


Supplementary Figure 14: **Map of plasmid C163.** Fluorescence reporter plasmid for the majority wins cyan strain. See Supplementary Tables 3 and 4 below for details.

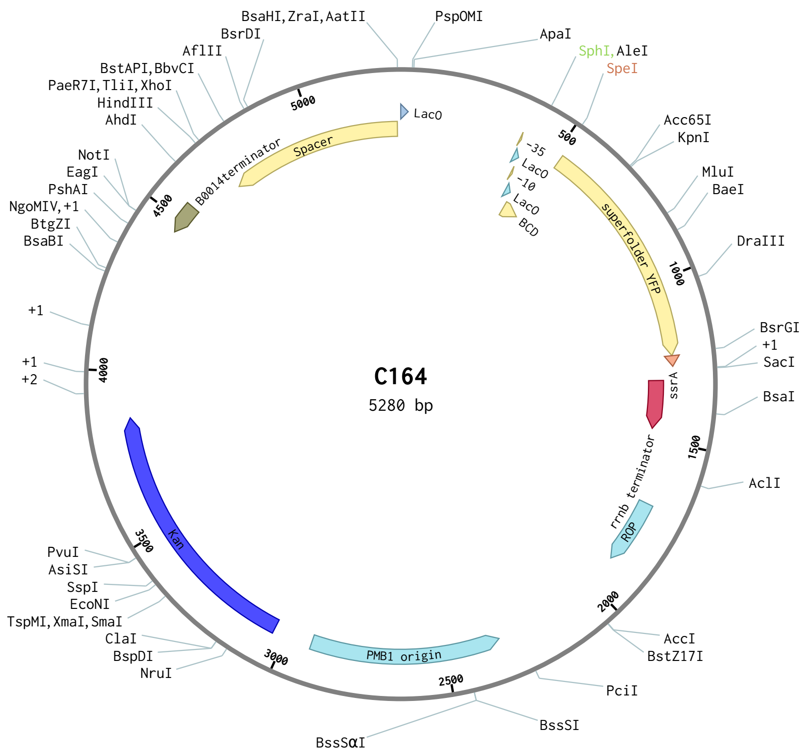


Supplementary Figure 15: **Map of plasmid C164.** Fluorescence reporter plasmid for the majority wins yellow strain. See Supplementary Tables 3 and 4 below for details.

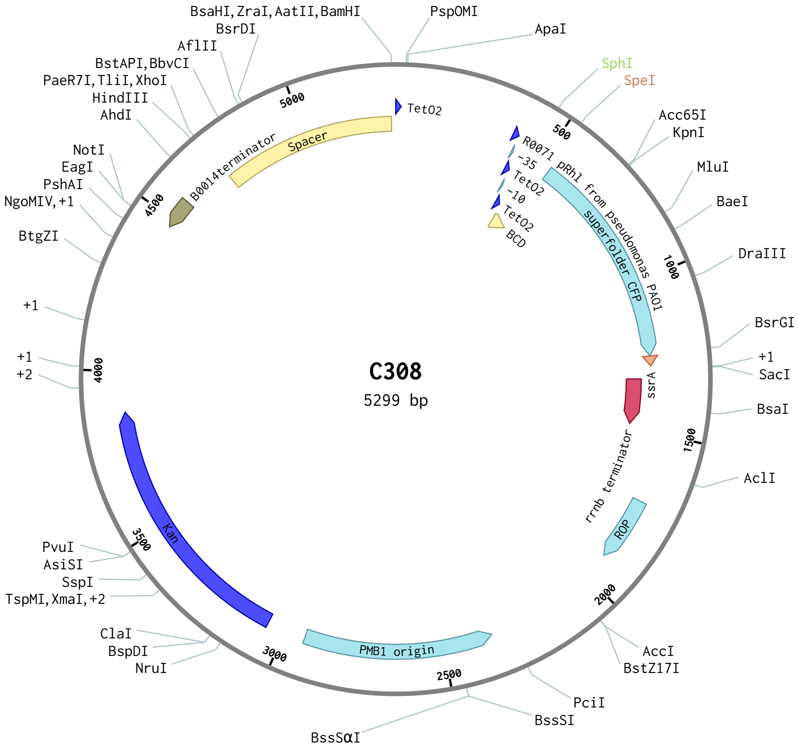


Supplementary Figure 16: **Map of plasmid C308.** Fluorescence reporter plasmid for the minority wins cyan strain. See Supplementary Tables 3 and 4 below for details.

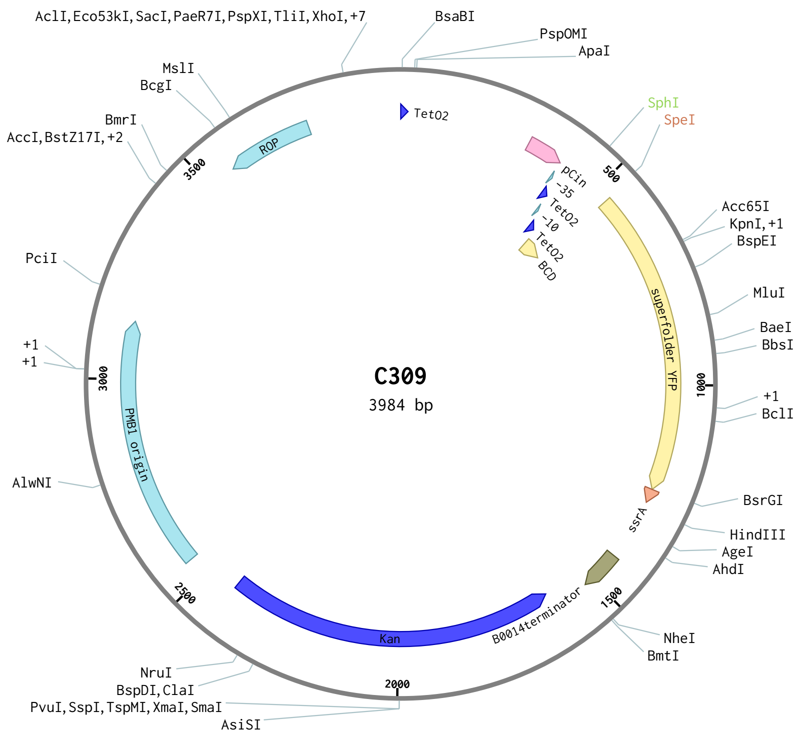


Supplementary Figure 17: **Map of plasmid C309.** Fluorescence reporter plasmid for the minority wins yellow strain. See Supplementary Tables 3 and 4 below for details.

| Strain | Plasmid | Gene Cassette | Resistance | Ori |
|---|---|---|---|---|
| Cyan strain plasmid 1 | C331 | plac–rhlI–LAA | specR | p15A |
| Cyan strain plasmid 2 | C163 | plac–sfCFP–LAA | kanR | pMB1 |
| Cyan strain plasmid 3 | C335 | pcin–rbsR-L–LAA | chlorR | pSC101 |
| Yellow strain plasmid 1 | C332 | plac–cinI–LAA | specR | p15A |
| Yellow strain plasmid 2 | C164 | plac–sfYFP–LAA | kanR | pMB1 |
| Yellow strain plasmid 3 | RA035 | prhl–lacI-11–LAA | chlorR | pSC101 |

Supplementary Table 3: **Majority Wins Plasmids.** The engineered *lac* promoters [1] and modified bicistronic design ribosome binding sites [2] drive the expression of *RhlI* (ATCC #47085), *CinI* (ATCC #10004), *sfcfp*, or *sfyfp* with standard LAA ssrA degradation tags [3]. The pMB1 plamids also contain an ROP element that reduces the copy number [4]. The engineered *cin* and *rhl* promoters [1] and modified bicistronic design ribosome binding sites [2] drive the expression of *RbsR-L* chimeric repressor and *LacI-11* dimeric repressor [5] with standard LAA ssrA degradation tags [3]. Both repressor plasmids also include the *AiiA* gene for QS molecule turnover [6] under the control of an engineered xylose inducible promoter [1] and bicistronic design ribosome binding site [2] with standard LAA ssrA degradation tags [3]. Each set of plasmids was transformed into a BW25113 derivative $\Delta araC$ $\Delta lacI$ $\Delta sdiA$ strain with *cinR* and *rhlR* inserted at the attB site under constitutive promoter (CY027) [7].

| Strain | Plasmid | Gene Cassette | Resistance | Ori |
|---|---|---|---|---|
| Yellow strain plasmid 1 | C331 | plac–rhlI–LAA | specR | p15A |
| Yellow strain plasmid 2 | C309 | pcin–sfYFP–LAA | kanR | pMB1 |
| Yellow strain plasmid 3 | C335 | pcin–rbsR-L–LAA | chlorR | pSC101 |
| Cyan strain plasmid 1 | C332 | plac–cinI–LAA | specR | p15A |
| Cyan strain plasmid 2 | C308 | prhl–sfCFP–LAA | kanR | pMB1 |
| Cyan strain plasmid 3 | RA035 | prhl–lacI-11–LAA | chlorR | pSC101 |

Supplementary Table 4: **Minority Wins Plasmids.** The engineered *lac* promoters [1] and modified bicistronic design ribosome binding sites [2] drive the expression of *RhlI* (ATCC #47085) and *CinI* (ATCC #10004) with standard LAA ssrA degradation tags [3]. The engineered *cin* and *rhl* promoters [1] and modified bicistronic design ribosome binding sites [2] drive the expression of *RbsR-L* chimeric repressor, *LacI-11* dimeric repressor [5], *sfcfp*, or *sfyfp* with standard LAA ssrA degradation tags [3]. The pMB1 plamids also contain an ROP element that reduces the copy number [4]. Both repressor plasmids also include the *AiiA* gene for QS molecule turnover [6] under the control of an engineered xylose inducible promoter [1] and bicistronic design ribosome binding site [2] with standard LAA ssrA degradation tags [3]. Each set of plasmids was transformed into a BW25113 derivative $\Delta araC$ $\Delta lacI$ $\Delta sdiA$ strain with *cinR* and *rhlR* inserted at the attB site under constitutive promoter (CY027) [7].

| Plasmid | 5' Sequencing Primer | 3' Sequencing Primer |
|---|---|---|
| C331 (RhlI) | AGTGCGCTCGAGCTTCCC | GATCAGTTGGAAGAATTTGTCCACT |
| C332 (CinI) | AGTGCGCTCGAGCTTCCC | GATCAGTTGGAAGAATTTGTCCACT |
| C335 (RbsR-L) | TCCCAACCTTACCAGAGGGC | TTGCGGGCAACTTCAGCA |
| C335 (AiiA) | GAACGGTTTCACTAGGAAGCAAG | TAGTGACCTGTTCGTTGCAACA |
| RA035 (LacI-11) | TCCCAACCTTACCAGAGGGC | TTGCGGGCAACTTCAGCA |
| RA035 (AiiA) | GAACGGTTTCACTAGGAAGCAAG | TAGTGACCTGTTCGTTGCAACA |
| C163 (sfCFP) | GAACGGTTTCACTAGGAAGCAAG | TATGGATGCGGCGGGAC |
| C164 (sfYFP) | GAACGGTTTCACTAGGAAGCAAG | TATGGATGCGGCGGGAC |
| C308 (sfCFP) | GAACGGTTTCACTAGGAAGCAAG | TATGGATGCGGCGGGAC |
| C309 (sfYFP) | TATGGATGCGGCGGGAC | GCCAGTGTGAGACAGCGGTGCGGAC |

Supplementary Table 5: **Sequencing Primers.** The primers used to sequence each plasmid insert.

# References


[1] Y. Chen, J. M. L. Ho, D. L. Shis, C. Gupta, J. Long, D. S. Wagner, W. Ott, K. Josić, and M. R. Bennett. Tuning the dynamic range of bacterial promoters regulated by ligand-inducible transcription factors. *Nat. Commun.*, 9:64 (2018).

[2] Vivek K. Mutalik, Joao C. Guimaraes, Guillaume Cambray, Colin Lam, Marc Juul Christoffersen, Quynh Anh Mai, Andrew B. Tran, Morgan Paull, Jay D. Keasling, Adam P. Arkin, and Drew Endy. Precise and reliable gene expression via standard transcription and translation initiation elements. *Nat. Methods*, 10(4):354–360 (2013).

[3] Jens Bo Andersen, Claus Sternberg, Lars Kongsbak Poulsen, Sara Petersen Bjørn, Michael Givskov, and Søren Molin. New Unstable Variants of Green Fluorescent Protein for Studies of Transient Gene Expression in Bacteria New Unstable Variants of Green Fluorescent Protein for Studies of Transient Gene Expression in Bacteria. *Appl. Environ. Microbiol.*, 64(6):2240–2246 (1998).

[4] R. M. Lacatena, D. W. Banner, L. Castagnoli, and G. Cesareni. Control of initiation of pMB1 replication: Purified rop protein and RNA I affect primer formation in vitro. *Cell*, 37(3):1009–1014 (1984).

[5] Sarah Meinhardt, Jr Manley, Michael W., Nicole A. Becker, Jacob A. Hessman, III Maher, L. James, and Liskin Swint-Kruse. Novel insights from hybrid LacI/GalR proteins: family-wide functional attributes and biologically significant variation in transcription repression. *Nucleic Acids Research*, 40(21):11139–11154 (2012).

[6] Tal Danino, Octavio Mondragón-Palomino, Lev Tsimring, and Jeff Hasty. A synchronized quorum of genetic clocks. *Nature*, 463(7279):326–330 (2010).

[7] Ye Chen, Jae Kyoung Kim, Andrew J Hirning, Krešimir Josić, and Matthew R Bennett. Emergent genetic oscillations in a synthetic microbial consortium. *Science*, 349(6251):986–989 (2015).
