# Supplementary material for: Majority sensing in synthetic microbial consortia
Source: Nat Commun. 2020 Jul 21;11:3659. doi: 10.1038/s41467-020-17475-z (PMC7374166; doi:10.1038/s41467-020-17475-z)
Supplement: Supplementary file 12 — Description of Additional Supplementary Files [file 41467_2020_17475_MOESM12_ESM.pdf]

Title: Supplementary Video 1: Majority Wins1.
Description: Video for the majority wins consortium data presented in Fig. 6. Trap begins with cyan strain in the majority and fluorescent. As the yellow strain begins to dominate the trap, the yellow cells become fluorescent and eventually shut off fluorescence in the cyan strain.

Title: Supplementary Video 2: Minority Wins1.
Description: Video for the minority wins consortium data presented in Fig. 6. Trap begins with cyan strain in the minority and fluorescent. As the cyan strain begins to dominate the trap, the cyan cells reduce in fluorescence and eventually turn on fluorescence in the yellow strain.

Title: Supplementary Video 3: Majority Wins2.
Description: Video for the top left replicate of the majority wins consortium data presented in Fig. 9. Trap begins with cyan strain in majority and fluorescent. As the yellow strain begins to dominate the trap, the yellow cells become fluorescent and eventually shut off fluorescence in the cyan strain. Then the cyan strain becomes majority again, turning on fluorescence in the cyan strain and turning off fluorescence in the yellow strain.

Title: Supplementary Video 4: Majority Wins3.
Description: Video for the middle left replicate of the majority wins consortium data presented in Fig. 9. Trap begins with cyan strain in majority and fluorescent. As the yellow strain begins to dominate the trap, the yellow cells become fluorescent and eventually shut off fluorescence in the cyan strain. There is even loss of the cyan strain completely.

Title: Supplementary Video 5: Majority Wins4.
Description: Video for the bottom left replicate of the majority wins consortium data presented in Fig. 9. Trap begins with cyan strain in majority and fluorescent. As the yellow strain begins to dominate the trap, the yellow cells become fluorescent and eventually shut off fluorescence in the cyan strain.

Title: Supplementary Video 6: Minority Wins2.
Description: Video for the top right replicate of the minority wins consortium data presented in Fig. 9. Trap begins with cyan strain fluorescent, but as the yellow strain becomes minority, cyan cells become non-fluorescent and the yellow cells become fluorescent.

Title: Supplementary Video 7: Minority Wins3.
Description: Video for the middle right replicate of the minority wins consortium data presented in Fig. 9. Trap begins with cyan strain fluorescent, but as the yellow strain becomes minority, cyan cells become non-fluorescent and the yellow cells become fluorescent.

Title: Supplementary Video 8: Minority Wins4.
Description: Video for the middle right replicate of the minority wins consortium data presented in Fig. 9. Trap begins with cyan strain fluorescent, but as the yellow strain becomes minority, cyan cells become non-fluorescent and the yellow cells become fluorescent. There is even loss of the cyan strain completely.
